# Knowledge, practice and associated factors towards intravenous cannula-related infection prevention among nurses working at Northwest Amhara Regional State Comprehensive Specialized Hospitals, Ethiopia

**DOI:** 10.1186/s12912-024-01737-y

**Published:** 2024-03-11

**Authors:** Alemwork Dessalegn, Mohammed Seid Ali, Senay Yohannes, Yeshimebet Tamir, Sileshi Mulatu, Ashenafi Zewdie

**Affiliations:** 1 Department of adult Health Nursing, school of Health Science, college of medicine and health science, Bahir Dar University, PO Box 79, Bahir Dar, Ethiopia; 2 Department of Pediatrics and child health nursing, college of medicine and health science, University of Gondar, Gondar, Ethiopia; 3 Departments of Surgical Nursing, college of medicine and health science, University of Gondar, Gondar, Ethiopia; 4 Department of Pediatrics and child health nursing, college of medicine and health science, Bahir Dar University, Bahir Dar, Ethiopia; 5 Department of Nursing and Midwifery, Wollega University, Institute of Health Science, Wollega, Ethiopia

**Keywords:** Hospital, Infection prevention, Intravenous, Knowledge, Nurses, Practice

## Abstract

**Background:**

Intravenous cannula-related infections are one of the leading causes of healthcare-associated infections. It leads to morbidity and mortality in hospitalized patients. Nurses play a significant role in the prevention of these infections. Whereas in Ethiopia, there is limited information and published studies done on nurses’ knowledge, practice, and associated factors and also most of other available studies done only the magnitude it lack associated factors. The purpose of this study was to assess nurses’ knowledge, practice, and associated factors toward intravenous cannula-related infection prevention.

**Methods:**

An institution-based cross-sectional study was conducted at Northwest Amhara Regional State Comprehensive Specialized Hospitals on May 1-30/2022. By using single population proportion formula the sample size was determined; we used a 50% proportion value (0.05), and 95% Confidence Interval 5% margin of error. A simple random sampling method was used to select 423 nurses. The data were collected by using structured pretested self-administered questionnaires. Then coded, and enter into epi-data version 4.6 and exported into the statistical package for social science version 23 for cleaning and analyzing the data. Data were presented by texts, tables, and figures. A binary logistic regression model was used to assess the association between variables. Based on the adjusted odds ratio, variables having a *p*-value less than 0.05 with a 95% confidence interval were used to state associated with the outcome variables.

**Results:**

A total of 412 nurses participated in this study with 97.4% response rate. The participants had good knowledge and practice in proportions of (54. 9%) and (53. 4%) respectively.

Being male, working wards/units, having training, and a higher educational level were factors that were significantly associated to having good knowledge. Working wards/units, having good knowledge, training, and access to guidelines were significantly associated with performing good practice.

**Conclusion:**

The finding of this study revealed that nearly half of the nurses had poor knowledge and practice in intravenous cannula-related infection prevention. As a result, hospital administrators and other concerned stakeholders better to prepare and ensure that guidelines are available, provide training, and develop the educational levels of nurses.

## Background

An intravenous (IV) cannula is an invasive procedure performed on hospitalized patients to administer drugs, fluids, and nutrients, and also take a blood sample intravenously either centrally or peripherally [1, 2]. More than 90% of hospital-admitted patients take IV medications [3]. However, it has its own complication such as phlebitis, pain, sepsis, venous thrombosis, hemorrhage, and infiltration [4–7]. These complications are mostly occurred due to poor healthcare practices during insertion, maintenance, and removal of the intravenous cannula [8–10]. Around 98.8% of intravenous cannula-related infections are preventable by nurses [11]. Even if Central Disease Control (CDC) develops a guideline for the prevention of infection related to IV cannula [12]. Still, the problem is a major public health problem, and it affects the health of the patient, and the quality of health care [13].

In the world, according to the International Nosocomial Infection Control Consortium (INICC) report around 1,789 patients develop intravenous cannula-related infections among 743,508 patients having IV cannula [14]. Its mortality rate increased by 18.9% & length of hospital stay by 6.46% [15]. In the United States of America, around 153 patients develop intravenous cannula-related infections among 85,063 patients having IV cannula each year [16]. It increased the death rate by 3%, lengths of stay in hospital by 2%, and also hospitalization costs by $3,886 [13]. In Southwest Asia, due to poor nursing care, seven times increase the incidence of intravenous cannula-related infection [17]. In Africa, around 40% of patients develop an intravenous cannula-related infection. It affects the health of the patient, increase length of hospital stay, and health care costs [14, 18]. Different studies showed that in Ethiopia around 84% and 61.1% of intravenous cannula-related infections are occurred due to poor nursing care practice [19, 20].

In the healthcare setting, more than 71% of intravenous cannulas were inserted and cared for by nurses [21]. Therefore, nurses play a significant role in the prevention of intravenous cannula-related infections. By applying intervention strategies like frequently assessing and monitoring the venous cannula site, preparing necessary equipment, selecting appropriate anatomical insertion sites, and also keeping sterility technics during insertion, medication administration, and removal [22, 23]. To perform those tasks and provide the patient high-quality care, it needs good knowledge and practice. [24]. Although studies showed that below half of the nurses had good knowledge and practice for instance in Egypt, 50.6%, 7.4% Nepal, 49.1%, 33.9%, and India, 35%, 33%, knowledge, and practice respectively [25–27].

Factors such as educational status, work experience, training towards intravenous cannula-related infection prevention, sex, and age of nurses were contributing factors to the nurse’s knowledge and practice towards intravenous cannula-related infection prevention [26–30].

Continuous quality improvements programs like education, training, and adherence to standardized guidelines towards intravenous cannula-related infection preventions are possible solutions to reduce IV cannula related infections. They increase the nurses’ knowledge, practices and also reduced the incidence of infection rate [31–34].

Most of the studies done only the magnitude of nurses' knowledge and practice but, this study includes the factors that affect the nurse's knowledge, and practice [3, 35–39]. And also in Ethiopia, there is limited information and published studies done on nurses’ knowledge, practice, and associated factors toward IV cannula-related infection prevention. Therefore, the aim of this study conducted to assess the nurse’s Knowledge, Practices, and associated factors regarding intravenous cannula-related infection prevention among nurses working at Northwest Amhara Regional State Comprehensive Specialized Hospital, Northwest Ethiopia.

## Method and material

### Study setting

The study was conducted in Northwest Amhara Regional State Comprehensive Specialized Hospitals.

It is situated in Ethiopia's northwest region. There are 5 comprehensive specialty hospitals. 1,756 nurses are found. Those are Debre Markos comprehensive specialized hospital is found in East Gojjam Zone Debre Markos town, which is located 300 km away from Addis Ababa, North West Ethiopia, and 265 km from Bihar Dar, the capital city of Amhara regional state. Tibebe Gione and Felege Hiwote Comprehensive Specialized Hospitals are found in Bahir Dar city, which is located 565 km away from Addis Ababa, University of Gondar Comprehensive Specialized Hospital is found in the Gondar town, which is located Northwest direction of Ethiopia, 727 km away from Addis Ababa, and 175 km from Bahir Dar, Debre Tabor Comprehensive Specialized Hospital is found in South Gondar 667 km away from Addis Ababa, and 102 km away from Bahir Dar.

### Study design and period

An institutional-based cross-sectional study was conducted from May 01–30/2022.

### Source and study populations

All nurses who were working at Northwest Amhara Regional State Comprehensive Specialized Hospitals were the source population and those nurses who were available during the data collection period were the study population.

### Study unit

Individual nurses who are randomly selected were our study unit

### Eligibility criteria

All nurses who were working in Northwest Amhara Regional State Comprehensive Specialized Hospitals, Northwest Ethiopia, available during the data collection period were included in the study.

### Sample size and sampling procedure

#### Sample size determination

The sample size was calculated by using the single population proportion formula:

n = (Zα/_2_)^2^ p (1-p)/d^2^ from the formula

n: denotes sample size,

Z: value of standard normal distribution at 95% CI (Z =1.96), (Z _α/2_)^2^= 3.84

P: is the proportion in the previous study but there was no previous study done in Ethiopia among staff nurses using *p*-value 50%= 0.5

d: is the level of standard error 95% CI 5%, ( d^2^ ) = 0.0025

n= (3.84)*0.5(1-0.5 / 0.0025 = 384.4

By adding a 10 % (38.4 nurses) non-response rate the final sample size was 423.

#### Sampling procedures

**Fig. 1:**
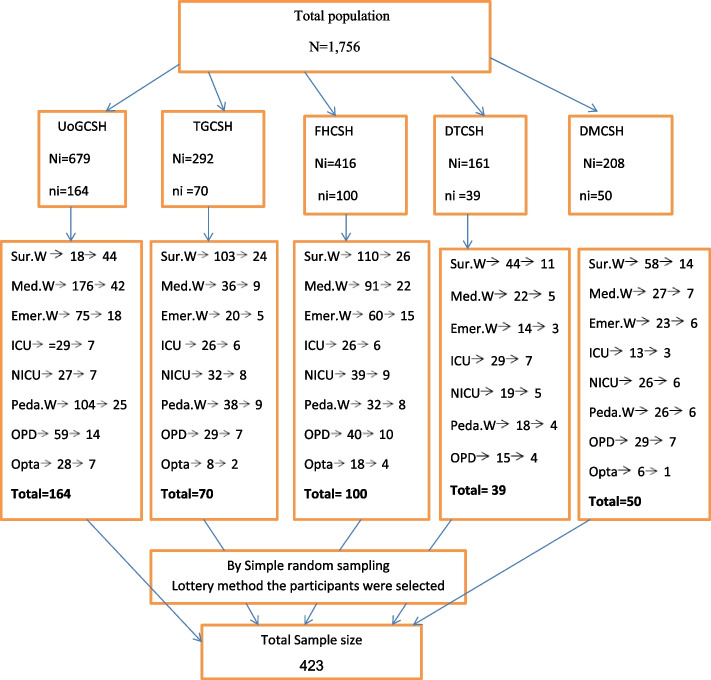
Schematic presentation of sampling procedure of nurses’ knowledge, practice, and associated factors on intravenous cannula related infection prevention in Northwest, Amhara regional state comprehensive specialized hospitals. Where; UoGCSH University of Gonder comprehensive Specialized Hospital. TGCSH Tibebe Gione Comprehensive Specialized Hospital. FHCSH Felege Hiwote comprehensive Specialized Hospital. DTCSH Debre Tabor comprehensive Specialized Hospital. DMCSH Debre Markos comprehensive Specialized Hospital. n total calculated sample size N total population for all hospitals, Ni total population from each hospital, ni sample size from each hospital. Sur.W Surgical Ward Med.W Medical Ward. Emer.W Emergency Ward ICU Intensive care unit. Peda.W Pediatrics Ward OPD Out Patient Department. Opta.C Ophthalmic clinic

Among one thousand seven hundred fifty six (1,756) nurses four hundred twenty three (423) nurses were selected by using simple random sampling method. Participants proportionally allocate by using proportional allocation formula from all Northwest Amhara Regional State-Comprehensive Specialized Hospitals. And also again it was proportionally allocated based on there working units/wards of each hospital. Finally, by using a simple random sampling (lottery method), the participants were selected from each study unit/ward (Fig. 1).

### Operational definitions

#### Good knowledge

Participants were considered to have a good knowledge if they score the median score or above, on the knowledge questions of IV cannula-related infection prevention

#### Poor knowledge

Participants were considered to have a poor knowledge if they score below the median score, on the knowledge questions of IV cannula-related infection prevention.

#### Nurse to patient ratio/day

Normal standard nurse to patient ratios per day are as follows:

Ward nurses: 1:5, Emergency nurses: 1:3, Intensive Care Unit nurses: 1:1.

To say having worked over load nurses provide patient care more than the normal standard/ day.

#### Good practice

Participants were considered have a good practice if they score the median score or above, on the practice questions of IV cannula-related infection prevention.

#### Poor practice

Participants were considered to have a poor practice if they score below the median score, on the practice questions of IV cannula-related infection prevention.

### Data collection tool and procedure

Self-administered close-ended questionnaires were used to collect the data related to the knowledge and practice of nurses on IV cannula-related infection prevention; the tool has four parts, which include socio-demographic characteristics, institutional, work, and other related factors. The questionnaires were adopted from published studies and guidelines [12, 29, 40]. The data was collected from all Amhara regional state comprehensive specialized hospital by 5 trained BSCnurses for data collector, and 5 trained MSCnurses for supervisor. Upon the data collection, the data collector provides appropriate information about the study to the participants and instructs them to full-fill the questionnaire independently to maintain the transmission of information between the participants and also informed that their names, and personal identity were not mentioned and confidentiality is kept to respond to honest responses. Then informed consent was obtained to assure their willingness to participate in this study.

### Data quality control and management

The questionnaire was pre-tested two weeks before the actual data collection period on 22 (5%) nurses in Dessie Comprehensive Specialized Hospital. The reliability of the tool was tested by Cronbach’s Alpha test the value was 0.812 and 0.804 for knowledge and practice respectively, and also the validity of the tool was tested by the face validity method. The principal investigator provided one day’s training for data collectors and supervisors about the objectives of the study, questionnaire, data collection procedure, and also the rights of the participants three days before the actual day of the data collection period. All data were checked for completeness and consistency on the data collection day, before data entry and during analysis.

### Data processing and analysis

The collected data were first checked manually by the supervisor and principal investigator for completeness and consistency during the data collection period and rechecked before data entry. Then, coded, and enter into the Epi-data version 4.6 and exported into the Statistical Package for Social Science (SPSS) version 23 for cleaning the data and analysis. Descriptive statics such as central tendency, frequency, and percentage were presented by texts, tables, and figures. Model fitness was checked by using the Hosmer-Lemeshow goodness of fit test its value was 0.867 and 0.352 for knowledge and practice respectively. Multicollinearity was cheeked by using the variance inflation factor (VIF) its value 1.025-1.99, and 0.526-0.976, 1.026-1.99, and tolerance its value 0.526-0.964 for knowledge, and practice respectively. A binary logistic regression model was used to see the association between dependent and independent variables. Due to the limited number of independent variables, we did not choose the variables by using bivariable analysis. All independent variables were directly entered into multivariable analysis, adjusted odds ratios were calculated, and variables with a *p*-value of 0.05 or less at a 95% confidence interval were deemed significant to the outcome variables.

## Results

### Socio demographic characteristic of nurses

From a total of 423 nurses, 412 nurses participated in this study with a response rate of 97.4%. Among the total participants, half (208 (50.5%)) were male. The median age of the respondents was 30 years with a standard deviation (±5.368), and the minimum and maximum ages of the respondent were 22 and 56 respectively. Most of the participants 345(83.7%) have a bachelor's degree and also two- third (274(66.7%)) of participants were married (Table 1).

**Table 1:** Socio-demographic characteristics of study participants working at Northwest Amhara regional state comprehensive specialized hospitals, 2022 (*n*=412)

Variables	Category	frequency	Percentage (%)
Age(years)	20-29	165	40
	30-39	208	50.5
	≥ 40	39	9.5
Sex	Male	208	50.5
	Female	204	49.5
Marital status	Single	115	27.9
	Married	274	66.5
	Divorced& widowed	23	5.6
Educational status	Diploma	19	4.6
	Degree	345	83.7
	Masters	48	11.7

### Institutional, work and other related factors on prevention IV cannula-related infection

One-third (137 (33.3%)) of participants reported that there is availability of guidelines in the hospital. More than two-thirds (298(72.3%)) of participants are having work overload. Among the participants, 112(27.2%) are working in the surgical ward, more than half (240 (58.3%)) of participants read guidelines for IV cannula-related infection prevention, one-fourth (104 (25.2%)) of nurses were trained on IV cannula-related infection prevention. More than one-third (152(36.9%)) of the participant were 6-9 years’ of work experience (Table 2).

**Table 2:** Institutional and other related factors about a study participant in Northwest Amhara regional state comprehensive specialized hospitals, 2022 (*n*=412)

Variables	Category	frequency	Percentage (%)
Availability of guideline	Yes	137	33.3
	No	275	66.7
Availability of written policy	Yes	67	16.3
	No	345	83.7
Availability of an organized committee	Yes	90	21.8
	No	322	78.2
Work over Load	Yes	298	72.3
	No	114	27.7
Working ward/units	Surgical	112	27.1
	Medical	84	20.4
	Emergency	48	11.7
	ICU	29	7
	Pediatrics	46	11.2
	NICU	40	9.7
	OPD	41	10
	nurses working in ophthalmic clinic	12	2.9
Read guidelines/review related literatures	Yes	240	58.3
	No	172	41.7
Training	Yes	104	25.2
	No	308	74.8
Work experience (in years)	≤ 5	125	30.3
	6-9	152	36.9
	≥ 10	135	32.8

### Nurses’ knowledge towards prevention of IV cannula-related infection

Among 412 study participants 226(54.9%), 95% CI (50.2, 59.5) of respondents had good knowledge of intravenous cannula-related infection prevention (Table 3).

**Table 3:** Nurses’ knowledge on intravenous cannula-related infection prevention working in Northwest Amhara regional state comprehensive specialized hospitals, 2022 (*n*=412)

Knowledge questions towards IV cannula-related infection prevention	Correct answer (True/False)	Response	
		Correctly answered (Yes)	Incorrectly answered (No)
The cannula gauge 14-20G suitable for adult patients, 22-24G suitable for pediatrics patient, to use for peripheral intravenous cannula insertion.	T	310 (75.2%)	102(24.8%)
The upper or lower extremities for adult and also the scalp (in neonates or young infants) can be used as peripheral intravenous cannula insertion site.	T	343 (83.3%)	69 (16.7%)
Peripheral IV cannula can be used only for 48-72 hours if no signs and symptoms of complication.	T	276 (67%)	136 (33%)
Phlebitis is the most identifiable infection related to peripheral intravenous cannula.	T	308 (74.8%)	104 (25.2%)
The environment situations will not be influence the risk of infection related to intravenous cannula.	F	267 (64.8%)	145 (35.2%)
Hand hygiene before procedure for IV cannula insertion is important in order to prevent infection	T	369 (89.6%)	43 (10.4%)
Maintaining aseptic technique only during insertion of intravenous cannula prevent infection occur.	F	166 (40.3%)	246 (59.7%)
Wearing non-sterile gloves during insertion of intravenous cannula are advisable if the access site is not touched after the application of skin antiseptics.	T	159 (38.6%)	253 (61.4%)
Skin preparations at insertion site are require before intravenous cannula inserted.	T	337 (81.8%)	75 (18.2%)
Increase attempts for cannula insertion will not increase the risk of infection.	F	246 (59.7%)	166 (40.3%)
Try again at the same place after the third failed venipuncture Attempt is recommended.	T	99 (24%)	313 (76%)
It is best to not use small, near veins at the arterial sites, joints, And hardened veins.	T	289 (70.1%)	123 (29.9%)
Central venous cannula insertion predisposes patients to higher risk of blood stream infections.	T	289 (70.1%)	123 (29.9%)
Patients with intravenous cannula are on risk of nosocomial infection.	T	221 (53.6%)	191 (46.4%)
Changing IV sets used to administer blood, blood product, or fat emulsion within24 hours of initiating infusion is not recommended.	F	202 (49%)	210 (51%)
IV cannula has to be flushed with Normal Saline following any intravenous Medications.	T	257 (62.4%)	155 (37.6%)
The insertion sites of the cannula on the lower extremity were the same infection risks as compared to the upper extremities.	F	186 (45.1%)	226 (54.9%)
Utilizing transparent dressing is helpful for recognizing the early infection signs.	T	285 (69.2%)	127 (30.8%)
Cannula with a larger diameter is used to pump fluids and medications faster and in greater quantity.	T	283 (68.7%)	129 (31.3%)
Removing intravenous cannula immediately if not in use, will help to reduce risk of infection occur.	T	296 (71.8%)	116 (28.2%)
Routinely replacing central venous cannula without signs and symptoms of any complication do not prevent cannula-related infections	T	182 (44.2%)	230 (55.8%)
Use steel needles cannulas are recommended for the administration of fluids and medication.	F	165 (40%)	247 (60%)
Use subclavian site, rather than a jugular or a femoral site, for central venous access in adult patients is recommended.	T	193 (46.8%)	219 (53.2%)
Not use topical antibiotic ointment or creams on intravenous cannula insertion sites, except for dialysis patients is recommended.	T	234 (56.8%)	178 (43.2%)
Use routinely anticoagulant therapy to reduce the risk of cannula-related infections is advisable.	F	201 (48.8%)	211 (51.2%)
Avoid using the femoral vein for central venous access in adult patients is advisable.	T	241 (58.5%)	171 (41.5%)

### Nurses’ practice towards prevention of IV cannula-related infection

The result of this study revealed that 221(53.6%), 95% CI (48.8, 58.7) of the respondents had good practice of intravenous cannula related infection prevention (Table 4).

**Table 4:** Nurses’ practice on intravenous cannula-related infection prevention working in Northwest Amhara regional state comprehensive specialized hospitals, 2022 (*n*=412

Practice questions towards IV cannula-related infection prevention	Response		
	Always	Sometimes	Never
Maintain aseptic technique throughout IV cannula care.	271 (65.8%)	119(28.9%)	22 (5.3%)
Use sterile plaster to cover the cannula site.	116 (28.2%)	103 (25%)	193(46.8%)
Flush the IV cannula with normal saline following any intravenous medications.	135 (32.8%)	129 (31.3%)	148 (35.9%)
Aseptic technique cannot be ensured change the cannula immediately.	199 (48.3%)	152 (36.9%)	61 (14.8%)
Replace catheter site plaster if becomes damp, loosened, or visibly soiled.	159(38.6%)	163 (39.6%)	90 (21.8%)
Use sterile personal protective equipment’s during central Venus cannula insertion.	114 (27.7%)	96 (23.3%)	202 (49%)
Prepare the skin with an antiseptic solution before insertion.	230 (55.8%)	129 (31.3%)	53 (12.9%)
Leave the area to dry completely after applying the antiseptic solution.	253 (61.4%)	121 (29.4%)	38 (9.2%)
Assess daily intravenous cannula insertion sites.	184 (44.7%)	164 (39.8%)	64 (15.5%)
Document date, time, site, size, and name of nurse who insert cannula.	139 (33.7%)	95 (23.1%)	178(43.2%)
Encourage the patient to report any changes in their cannula site.	209 (50.8%)	125 (30.3%)	78 (18.9%)
Change the IV sets used to administer blood/blood product/ fat emulsion within 24 hours of initiating the infusion.	153 (37.1%)	122 (39.6%)	137 (33.3%)
Hand hygiene before intravenous cannula insertion.	155 (37.6%)	148(35.9%)	109 (26.5%)
Immediately Change peripheral intravenous cannula after 72 hours without any infection singe	135 (32.8%)	121 (29.3%)	156 (37.9%)
Use transparent plaster when securing the cannula.	117 (28.4%)	106 (25.7%)	189 (45.9%)
Educate the patient about the signs and symptoms of IV cannula related infection.	196 (47.6%)	160 (38.8%)	56 (13.6%)
Immediately change the cannula if there is sign of infection.	308 (74.4%)	76 (18.4%)	28(6.8%)

### Factors associated with nurses’ knowledge towards IV cannula-related infection prevention

Variables that were ≤ 0.05 *p*-value with 95% CI were significantly associated with the outcome variable.

According to this analysis had taken training, working ward/unit, sex, and educational level BSc degree and above were significantly associated with the knowledge of nurses.

Nurses who had taken training were 2.16 times more likely knowledgeable as compared to nurses who had not trained [AOR = 2.16, 95% CI (1.31, 3.57)]. Being male nurses 3.29 times more likely knowledgeable about IV cannula-related infection prevention as compared to female nurses [AOR = 3.29, 95% CI (2.14, 5.06)]. Nurses who have master, and bachelor's degrees were 3.81, and 3.65 times more likely knowledgeable about IV cannula-related infection prevention as compared to nurses who had diplomas [AOR = 3.81, 95% CI (1.07, 13.53)] and [AOR= 3.65, 95% CI (1.21, 11.05)] respectively. Nurses who worked in surgical 3.02, Pediatrics 2.83, and NICU 3.24 times more likely knowledgeable on IV cannula related infection prevention as compared to nurses who worked in the outpatient department [AOR= 3.02, 95% CI (1.34, 6.81)], [AOR= 2.83, 95% CI (1.08, 7.37)], [AOR = 3.24, 95% CI (1.19, 8.77)] respectively (Table 5).

**Table 5:** Bivariable and Multivariable analysis of factors associated with nurses’ knowledge towards prevention of intravenous cannula-related infection, Northwest Amhara regional state comprehensive specialized hospitals, 2022 (*n*=412)

Variables	knowledge		COR with (95% CI)	AOR with (95% CI)	*P*-value
	Good	Poor			
Age					
20-29	96(58.2%)	69(41.8%)	2.00 (0.98, 4.06)	1.63 (0.64, 4.19)	0.31
30-39	114(54.8)	94(45.2%)	1.74 (0.87, 3.49)	1.56 (0.69, 3.54)	0.29
≥40	16(41%)	23(59%)	1	1	1
**Sex**					
Male	146(70.2%)	62(29.8%)	3.65 (2.43, 5.49)	3.29 (2.14, 5.06)	0.00*
Female	80(39.2%)	124(60.8%)	1	1	1
Marital status					
Single	74(64.3%)	41(35.7%)	1	1	1
Married	144(52.6%)	130(47.4%)	0.61 (0.39,0.96)	0.61 (.37, 1.01)	0.05
Divorced & Widowed	8(34.8%)	15(65.2%)	0.21 (0.12,0.76)	0.39 (0.14, 1.08)	0.07
**Education**					
Diploma	5(26.3%)	14(73.7%)	1	1	1
Degree	191(55.4%)	154(44.6%)	3.47 (1.22,9.85)	3.65(1.21, 11.05)	0.02*
Masters	30(62.5%)	18(37.5%)	4.67(1.44,15.13)	3.81 (1.07, 13.53)	0.04*
Work experience					
≤5	75(60.0%)	50(40.0%)	1	1	1
6-9	87(57.2%)	65(42.8%)	0.89 (0.55,1.44)	0.98 (0.54, 1.77)	0.93
≥10	64(47.4%)	71(52.6%)	0.60 (0.37,0.98)	1.04 (0.41, 2.21)	0.90
Work over load					
Yes	157(52.7%	141(47.3%	0.73 (0.47, 1.13)	0.69 (0.42, 1.15)	0.15
No	69(60.5%)	45(39.5%)	1	1	1
**Working ward**					
OPD	17(41.5%)	24(58.5%)	1	1	1
**Surgical**	65(58.0%)	47(42.0%)	1.95 (0.95,4.04)	3.02 (1.34, 6.81)	0.01*
Medical	45(53.6%)	39(46.4%)	1.63 (0.77,3.47)	1.98 (0.86, 4.54)	0.11
Emergency	25(52.1%)	23(47.9%)	1.54 (0.66,3.56)	2.41 (0.95, 6.13)	0.07
ICU	17(58.6%)	12(41.4%)	2.00 (0.76,5.25)	2.68 (0.92, 7.82)	0.07
**Pediatrics**	27(58.7%)	19(41.3%)	2.01(0.85,4.72)	2.83 (1.08, 7.37)	0.03*
**NICU**	25(62.5%)	15(37.5%)	2.35 (0.96,5.74)	3.24 (1.19, 8.77)	0.02*
ophthalmic clinic					
Opta	5(41.7%)	7(58.3%)	1.01(0.27 ,3.72)	1.07 (0.25, 4.50)	0.93
**Training**					
Yes	70(67.3%)	34(32.7%)	2.01 (1.26,3.19)	2.16 (1.31, 3.57)	0.00*
No	156(50.6%)	152(49.4%)	1		
Read guideline					
Yes	135(56.3)	105(43.8)	1.14 (0.77, 1.69)	0. 83 (0.51, 1.34)	0.45
No	91(52.9)	81(47.1)	1	1	1
Avail. of guideline					
Yes	77(56.2)	60(43.8)	1.09 (0.72,1.64)	1.08 (0.63, 1.85)	0.78
No	149(54.2)	126(45.8)	1	1	1
Avail. of Written policy					
Yes	36(53.7)	31(46.3)	0.95(0.56, 1.60)	0.90 (0.47, 1.72)	0.75
No	190(55.1)	155(44.9)	1	1	1
Avail. of committee					
Yes	47(52.2)	43(47.8)	0.87 (0.55, 1.39)	0.79 (0.45, 1.39)	0.42
No	179(55.6)	143(44.4)	1	1	1

^*^Statistical significance at *p* value < 0.05 and CI didn’t include 1 with AOR, (*) variables which are significantly associated, 1 = for reference Category, Avail= Availability

### Factors associated with nurses’ practice towards IV cannula-related infection prevention

Variables that were ≤ 0.05 *p*-value with 95% CI were significantly associated with the outcome variable. According to this analysis having good knowledge, having taken training, availability of guidelines, and working wards/units were significantly associated with the practice of nurses. Nurses who have good knowledge were 2.55 times more likely to perform good practice towards IV cannula-related infection prevention as compared to nurses who do not have good knowledge [AOR = 2.55, 95% CI (1.67, 3.89)]. Nurses who had taken training were 2.12 times more likely good practice towards IV cannula-related infection prevention as compared to nurses who had not trained [AOR = 2.12, 95% CI (1.26, 3.57)]. Nurses who worked in the availability of guidelines in the ward/unit were 1.69 times more likely good practice as compared to nurses who worked not in the availability of guidelines in the ward/unit [AOR = 1.69, 95% CI (1.06, 2.68)]. Nurses who worked in the emergency (3.11), intensive care unit (3.98), pediatrics (4.88), and neonatal intensive care unit (4.69) times more likely to perform good practices towards IV cannula-related infection prevention as compared to nurses who worked in the outpatient department [AOR=3.11, 95% CI (1.22, 7.93)], [AOR= 3.98, 95% CI (1.36, 11.61)], [AOR = 4.88, 95% CI (1.83,13.05)], [AOR= 4.69, 95% (1.74,12.71)] respectively (Table 6).

**Table 6:** Bivariable and Multivariable analysis of factors associated with nurses’ practice towards prevention of intravenous-cannula related infection, Northwest Amhara regional state comprehensive specialized hospitals, 2022 (*n*=412)

**Variables**	**Practice**		**COR** (95% CI)	**AOR (95% CI)**	***P*-value**
	Good	Poor			
Marital status					
Single	63 (54.8%)	52 (45.2%)	1	1	1
Married	149 (54.4%)	125 (45.6%)	0.98 (0.64, 1.52)	1.23 (0.76, 2.00)	0.401
Divorced & widow	9 (39.1%)	14 (60.9%)	0.53(0.21,1.32)	0.65 (0.23, 1.83)	0.418
**Availability guideline**					
Yes	88 (64.2%)	49 (35.8%)	1.92(1.26, 2.92)	1.69 (1.06, 2.68)	0.027*
No	133 (48.4%)	142 (51.6%)	1	1	1
Avail. of written policy					
Yes	46 (68.7%)	21 (31.3%)	2.13 (1.22, 3.72)	1.29 (0.64, 2.61)	0.473
No	175 (50.7%)	170 (49.3%)	1	1	1
Avail. organized Commit.					
Yes	60 (66.7%)	30 (33.3%)	2.00 (1.23,3.26)	1.53 (0.85, 2.75)	0.152
No	161 (50.0%)	161 (50.0%)	1	1	1
**Working ward/unit:**					
OPD	13 (31.7%)	28 (68.3%)	1	1	1
Surgical	56 (50.0%)	56 (50.0%)	2.15(1.012, 4.58)	2.25 (0.99, 5.09)	0.051
Medical	42 (50.0%)	42 (50.0%)	2.15(0.98, 4.72)	2.11 (0.91, 4.91)	0.082
Emergency	28 (58.3%)	20 (41.7%)	3.02(1.26, 7.22)	3.11 (1.22, 7.93)	0.018*
ICU	18 (62.1%)	11 (37.9%)	3.52(1.30, 9.56)	3.98(1.36, 11.61)	0.012*
Pediatrics	32 (69.6%)	14 (30.4%)	4.92(1.98,12.22)	4.88 (1.83,13.05)	0.002*
NICU	27 (67.5%)	13 (32.5%)	4.47(1.76,11.37)	4.69 (1.74,12.71)	0.002*
ophthalmic clinic	5 (41.7%)	7 (58.3%)	1.54 (0.41, 5.78)	1.54 (0.38, 6.15)	0.543
Read guideline/review					
Yes	137 (57.1%)	103 (42.9%)	1.39(0.94, 2.07)	1.13 (0.68, 1.87)	0.650
No	84 (48.8%)	88 (51.2%	1	1	1
**Training**					
Yes	71 (68.3%)	33 (31.7%)	2.27 (1.42, 3.62)	2.12 (1.26, 3.57)	0.005*
No	150 (48.7%)	158 (51.3%)	1	1	1
**Knowledge**					
Good	147 (65.0%)	79 (35.0%)	2.82 (1.89, 4.21)	2.55 (1.67, 3.89)	0.000*
Poor	74 (39.8%)	112 (60.2%)	1	1	1
Age					
20-29	94(57%)	71(43%)	1.39 (0.69, 2.81)	1.34 (0.59, 3.03)	0.480
30-39	108(51.9%)	100(48.1%)	1.14 (0.57, 2.25)	1.04 (0.48, 2.26)	0.932
≥40	19(48.7%)	20(51.3%)	1	1	1
Education					
Diploma	9 (47.4%)	10(52.6%)	1	1	
Degree	189 (54.8%)	156 (45.2%)	1.35 (0.53, 3.39)	1.33 (0.44, 3.99)	0.615
Masters	23(47.9%)	25(52.1%)	1.022 (0.35, 2.96)	1.09 (0.29, 3.98)	0.895
Work experience					
<5	72 (57.6%)	53(42.4%)	1	1	1
6-9	81 (53.3%)	71 (46.7%)	0.84 (0.52, 1.35)	0.91 (0.50, 1.64)	0.753
≥10	68 (50.4%)	67 (49.6%)	0.75 (0.458, 1.22)	1.02 (0.49, 2.14)	0.958
Work over load					
Yes	160 (53.7%)	138 (46.3%)	1.01 (0.65, 1.55)	1.09 (0.66, 1.79)	0.732
No	61(53.5%)	53 (46.5%)	1	1	1
Sex					
Male	112(53.8%)	96(46.2%)	1.02 (0.69, 1.49)	0.69 (0.44, 1.11)	0.130
Female	109 (53.4%)	95 (46.6%)	1	1	1

^*^Statistical significance at *p* value < 0.05 and CI didn’t include 1 with AOR, (*) variables which are significantly associated, 1 = for reference Category, Avail= Availability

## Discussion

Intravenous cannula-related infections are the most common healthcare-acquired infection. It occurs mostly by poor healthcare practices [10]. The finding of this study showed that 54.9 % with a 95% CI (50.2, 59.5) of the study participants had good knowledge of intravenous cannula-related infection prevention. This finding is in line with a study conducted in Egypt that showed that 50.6% of nurses had good knowledge of IV cannula-related infection prevention [25]. Whereas this finding is higher than studies conducted in India 35% [27], and Yemen 21% [41]. This inconsistency might be due to socio-demographic differences [42], and the differences in results scoring those studies; the knowledge was scored as good, moderate, and poor in this study scored as good and poor knowledge and also in Yemen, there is socio-politically instability (war) due to this nurses might not upgrade their educational level, the findings indicate that more than half of the study participants held nursing diplomas, and there is a correlation between educational status and level of knowledge. Additionally, the timing of the Indian study differs from the current one by five years it might be due to the time difference develop technology for instance accessibility of the internet nurse's motivation to read related books and guidelines, and the participants were chosen using a consecutive sampling technique, whereas in this study using simple random sampling technique,

However, the result of this study was lower than the studies conducted in China, 83.5% [31], Nepal, 82.4% [35], and Malaysia,75.9% [36]. This inconsistency might be due to differences in the study setting; the study was conducted in Nepal and Malaysia in a single hospital, and in China ninety-one hospitals, whereas in this study the study was conducted in five hospitals, and also the difference might be due study units; in those studies participates were selected only in inpatient ward nurses whereas this study includes nurses working in all wards/units, and also the difference might be due to sampling technique the study done in Nepal census method of sampling technique was used whereas in this study simple random sampling technique was used and also the difference might be due to training the study showed that in Chain, around half of the nurses were trained towards IV cannula-related infection prevention whereas, in this study only twenty-five percent of nurses were trained. Nurses who received training increase their knowledge and practice on intravenous cannula-related infection prevention [32].

In multivariable logistic regression analysis, variables that were significantly associated with the knowledge of nurses towards IV cannula-related infection prevention were sex, educational level, training, and working wards/ units.

Nurses who had taken training were more likely knowledgeable as compared to nurses who had not trained. This finding is supported by studies conducted in Nepal, China, and Asia [26, 30, 37]. This could be updating the knowledge of nurses could have better knowledge they would easily understand basic principles and standards of practice and also motivates them to further read related books/literature [32, 43]. Being male nurses more likely knowledgeable about IV cannula-related infection prevention as compared to female nurses. This finding is supported by the study conducted in India [27]. The possible explanation for this finding might be the educational status of nurses in this study the majority of the master's nurses were male nurses and diploma nurses were female nurses. The result showed that participants who had master's nurses are more likely to have better knowledge than diploma nurses, this result might be mostly out-of-work females who spend their time in home-based activities for instance; food preparation, child caring, and home-based sanitation. Therefore, may not get time to upgrade their educational levels [44].

Nurses who have masters and bachelor's degrees were more likely knowledgeable about IV cannula-related infection prevention as compared to nurses who had diploma nurses. This finding is supported by the studies conducted in Malaysia and Iraq [29, 45]. This may be because nurses with higher education levels have acquired important knowledge, which has encouraged them to participate in courses on IV cannula-related infection prevention, directly or indirectly.

Nurses who worked in surgical, Pediatrics, and NICU were more likely knowledgeable on IV cannula-related infection prevention as compared to nurses who worked in the outpatient department. The possible justification for this might be the procedure is routinely performed in those wards/units; Nurses might be motivated to familiarize themselves with IV cannula-related infection prevention by reading related books and reviewing related literatures. The result of this study showed that in those wards/units, more than fifty percent of nurses read guidelines /review-related literatures whereas only seventeen percent of nurses working in OPD read guidelines /review-related literatures. Literature support that it is a positive relationship between knowledge and reading practice [43].

The finding of this study showed that 53.6% with a 95% CI [48.8, 58.7] of the study participants had good practices in intravenous cannula-related infection prevention. which is in line with a study conducted in Egypt which is 53.3% [38]. While this finding is higher than the studies conducted in India 33%, and Nepal 33.9% [26, 39]. The difference could be due to the difference in study designs those studies were done observational whereas this study was done by self-report, and the time of study those studies were done 5 and 3 years before this study. It might be due to time differences develop technology for instance accessibility of the internet is high nurses motivate to read related books/guidelines.

However, this study was lower than studies conducted in Malaysia which is 83.7% [36], and India which is 68% [45]. This difference might be due to socio-demographic differences, and also in an Indian study due to training the result showed that more than fifty percent of nurses were trained but in this study, only twenty-five percent of nurses were trained on IV cannula-related infection prevention. Nurses who received training increase their practice. The Malaysian study, included only inpatient ward nurses, whereas all nurses workings in the hospital were included in this study. Additionally, participants were selected using purposive sampling, whereas this study used simple random sampling.

In a multivariable logistic regression analysis, variables that were significantly associated with the practice of nurses toward IV cannula-related infection prevention were having good knowledge, training on IV cannula-related infection prevention, availability of guidelines, and working ward/ unit.

Nurses who had good knowledge were more likely to perform good practice towards IV cannula-related infection prevention as compared to nurses who had not good knowledge. This finding was supported by studies conducted in India and Egypt [37, 46]. This might be due to if the nurses have more knowledge of a particular activity they are more skillful in that activity and it increases their confidence to perform the activities. To provide holistic and continuous quality nursing care at every level of service delivery needs adequate knowledge [47].

Nurses who had taken training were more likely good practice towards IV cannula-related infection prevention as compared to nurses who had not trained. This finding was supported by studies conducted in Egypt, and Greek [1, 48]. The possible explanation for this finding could be the fact that training could upgrade the knowledge and skill of nurses. They would easily understand basic principles and standards of practice and implement them constantly [31].

Nurses working in wards/ units that have availability of guidelines is more likely to increase their good practice as compared nurses working in wards/units that were not availability of guidelines. The possible justification might be due to nurses who have worked in guidelines availability wards/units were more likely to know how to prevent this infection and get updated information which increases their practice of intravenous cannula-related infection prevention interventions [49].

Nurses who worked in the emergency, intensive care unit, pediatrics, and neonatal intensive care unit more likely to perform good practice towards IV cannula-related infection prevention as compared to nurses who worked in the outpatient department. This finding is supported by a study conducted in Italy [28]. This might be in those wards/units the procedure is routinely performed. It motivates nurses to know about intravenous cannula-related infection prevention methods also it might be nurses' more exposure to this procedure have a greater chance to know how to prevent IV cannula-related infection prevention from their own mistakes and also their Colleague’s [50].

Extensive efforts have been made to minimize the possible limitation of this study. Biases, especially those associated with participants’ practices, may exist in self-reported; therefore, participants may overstate their good practices. The cross-sectional nature of this study will make it unable to form a temporal relationship between the outcome and predictor variables. The study is also prone to social desirability bias which could lead to over/ underestimation of the study finding.

## Conclusion

The results of this study indicated that nearly half of the nurses had poor knowledge and practice in intravenous cannula-related infection prevention. Having a higher educational level, attending formal training, sex, and working in inpatient wards indicated that positive association with good knowledge. Whereas having good knowledge, attending formal training, having availability of guidelines, and also working in inpatient wards were found to be significantly associated with good practice in intravenous cannula-related infection prevention.

Based on this finding, the Ministry of Health and the Hospital administrators with the collaboration of other stakeholders have to be made to update the knowledge and practice of healthcare workers. Regarding intravenous cannula-related infection prevention activities by providing pre-service or in-service training, preparing and informing intravenous cannula-related infection prevention guidelines, developing educational level of nurses, and inpatient ward nurses better to share their knowledge and practice with outpatient department nurses in order to improve their knowledge and practice towards intravenous cannula-related infection prevention.

Researcher will be done further researches and also using qualitative research methods to assess practice of nurses towards intravenous cannula-related infection prevention.

## Data Availability

The datasets used and analyzed during the current study are available from the corresponding author (AD) upon reasonable request. Reuse of the data is permitted for non-commercial purposes. Contact details: Email: Alemwork.des@gmail.com.

## Abbreviations

AOR: Adjusted Odds Ratio
CI: Confidence Interval
COR: Crude Odds Ratio
DMCSH: Debre Markose Comprehensive Specialized Hospital
DTCSH: Debre Tabore Comprehensive Specialized Hospital
Emer.W: Emergency Ward
FHCSH: Felege Hiwot Comprehensive Specialized Hospital
ICU: Intensive Care Unit
IP: Infection Prevention
IRB: Institutional Review Bored
IV: Intravenous
Med.W: Medical Ward
NICU: Neonatal Intensive Care Unit
OPD: Out Patient Department
Peda.W: Pediatrics Ward
Sur.W: Surgical Ward
TGCSH: Tibebe Gione Comprehensive Specialized Hospital
UoGCSH: University of Gondar Comprehensive Specialized Hospital
